# Neural Dynamics of Emotional Salience Processing in Response to Voices during the Stages of Sleep

**DOI:** 10.3389/fnbeh.2016.00117

**Published:** 2016-06-14

**Authors:** Chenyi Chen, Jia-Ying Sung, Yawei Cheng

**Affiliations:** ^1^Institute of Neuroscience, National Yang-Ming UniversityTaipei, Taiwan; ^2^Department of Neurology, Wan Fang Hospital, Taipei Medical UniversityTaipei, Taiwan; ^3^Department of Neurology, School of Medicine, College of Medicine, Taipei Medical UniversityTaipei, Taiwan; ^4^Department of Rehabilitation, National Yang-Ming University HospitalYilan, Taiwan

**Keywords:** sleep, emotional salience, voices, mismatch negativity (MMN), rapid eye movement (REM)

## Abstract

Sleep has been related to emotional functioning. However, the extent to which emotional salience is processed during sleep is unknown. To address this concern, we investigated night sleep in healthy adults regarding brain reactivity to the emotionally (happily, fearfully) spoken meaningless syllables *dada*, along with correspondingly synthesized nonvocal sounds. Electroencephalogram (EEG) signals were continuously acquired during an entire night of sleep while we applied a passive auditory oddball paradigm. During all stages of sleep, mismatch negativity (MMN) in response to emotional syllables, which is an index for emotional salience processing of voices, was detected. In contrast, MMN to acoustically matching nonvocal sounds was undetected during Sleep Stage 2 and 3 as well as rapid eye movement (REM) sleep. Post-MMN positivity (PMP) was identified with larger amplitudes during Stage 3, and at earlier latencies during REM sleep, relative to wakefulness. These findings clearly demonstrated the neural dynamics of emotional salience processing during the stages of sleep.

## Introduction

Currently, the sleeping brain is considered an active processor that reacts to the external world (Perrin et al., 1999, 2002; Bastuji et al., 2002; Ruby et al., 2008). In such circumstances, we expect the sleeping brain to process emotions. However, the extent to which emotional salience is processed during the stages of sleep remains to be determined.

A large body of research, using similar paradigms to deliver sensory stimuli to sleeping vs. awaking subjects, had provided converging evidence to support the neural responsiveness during sleep (Coenen, 1995; Born et al., 2002; Coenen and Drinkenburg, 2002; Lavigne et al., 2004; Hennevin et al., 2007; Karakaş et al., 2007; Ibáñez et al., 2009). In particular, the auditory system continues to work during sleep (Atienza et al., 2001a). For instance, N400, a component of event-related potentials (ERP) elicited by the presentation of semantically unrelated information between two words or between a context and a word, corroborated the processing of semantic discrimination during stage 2 and rapid eye movement (REM) sleep (Brualla et al., 1998; Ibáñez et al., 2006). P3 was enhanced by the subject’s own name and K-complexes were evoked by all first names (Perrin et al., 1999, 2000). Sleeping subjects were substantially awakened faster by hearing their own names than other names (Oswald et al., 1960). Amygdala had a purported role of rapid, automatic and non-conscious processing of emotional and social stimuli (Pessoa and Adolphs, 2010; Tamietto and de Gelder, 2010). The subject’s own name relative to tones produced stronger activation in the left amygdala and prefrontal cortex during stage 2 (Portas et al., 2000). Even the learned representation of conditioned-fear, the initial neutral stimulus (conditioned stimulus, CS) acquired a behavioral significance through paring with a biologically relevant stimulus (unconditioned stimulus, US), was identified during sleep (Maho et al., 1991; Hennevin et al., 1993; Maho and Hennevin, 1999). The sleeping brain can discriminate relevant from irrelevant stimuli, particularly when the stimuli are significantly salient and intrinsically meaningful.

However, existing knowledge regarding emotional salience processing in the sleeping brain is mainly drawing from the observed modulation of a learned stimulus (the conditioned stimulus, CS; Maho and Hennevin, 1999), intrinsically meaningful stimulus (e.g., subject’s own names; Portas et al., 2000) or the comparisons of the behavioral and neuroimaging observations immediately before and after sleep (Yoo et al., 2007; Gujar et al., 2011a,b; van der Helm et al., 2011). The sensitivity to emotional facial expressions was reported to change after REM sleep (Gujar et al., 2011a). The amygdala activity involved in REM sleep was associated with emotional intensities of dreams (Maquet et al., 1996). Overnight sleep attenuated the amygdala activity in response to previously encountered emotional stimuli (van der Helm et al., 2011). Sleep deprivation enhanced the amygdala response to negative emotional stimuli (Yoo et al., 2007) and amplified the neural reactivity responsible for rewarding (Gujar et al., 2011b). Hence, the direct evidence to the processing of emotional salience during sleep is warranted (Hennevin et al., 2007). Specifically, to what extent are the neurophysiological indices of sleep functionally equivalent to their waking counterparts? Is the processing of emotional salience during sleep comparably efficient with that during wakefulness?

The passive oddball paradigm enables the investigation of automatic auditory processing during wakefulness and sleep, as indicated by neurophysiological indices of N1-P2 complex, mismatch negativity (MMN), and post-MMN positivity (PMP). The N1-P2 complex, a sensory processing index, has a time window of 100−300 ms during wakefulness (Doellinger et al., 2011), and shifts to approximately 70−225 ms during sleep (Nordby et al., 1996). The MMN, which reflects automatic discrimination of auditory changes in human auditory cortex (Näätänen et al., 2011), is identified during REM sleep (Nashida et al., 2000) as well as in Stage 1 and Stage 3 (Ruby et al., 2008). MMN in response to emotional syllables have been recently used to index emotional salience processing of voices at the preattentive stage (Cheng et al., 2012; Fan et al., 2013; Hung and Cheng, 2014). The MMN in response to meaningless syllables spoken with disgusted prosody generated cortical activity in the anterior insular cortex (Chen et al., 2014). Emotional MMN became atypical in individuals with empathy deficits (Hung et al., 2013; Fan and Cheng, 2014). Furthermore, testosterone had impact on emotional MMN (Chen et al., 2015), indicating the involvement of amygdala in the generation of early ERP components responsible for emotional perception (Sabatinelli et al., 2013). The PMP, a P3a-like wave recorded during transition to sleep and also during sleep (Cote, 2002), was elicited when the novel stimuli were sufficiently salient so as to intrude into consciousness (Putnam and Roth, 1990; Niiyama et al., 1994; Bastuji et al., 1995).

To elucidate the neural dynamics of emotional salience processing to vocal stimuli during sleep stages, this study used the passive oddball paradigm with deviants in the emotional syllables *dada* and correspondingly synthesized nonvocal sounds. We hypothesized that if the sleeping brain were able to process emotional salience *per se*, MMN and PMP, particularly during REM sleep, would respond to emotional syllables rather than nonvocal sounds. In contrast, if the sleeping brain were insensitive to emotions, MMN and PMP in response to emotional syllables would not be identified during sleep.

## Materials and Methods

### Participants

Twelve healthy subjects (6 females) aged 23–27 years (mean ± SD = 24 ± 1.3 years) volunteered to participate in this study. They were all self-reported good sleepers and were not using ongoing medications. All participants had normal peripheral hearing bilaterally (pure tone average thresholds <15 dB HL) and normal middle ear function at the time of testing. None had a history of neurological or psychiatric problems. The local Ethics Committee (Yang-Ming University Hospital) approved this study. In accordance with the Declaration of Helsinki, all participants provided informed consent and received instructions regarding all the experimental details, as well as their right to withdraw at any time. In addition, participants refrained from ingesting caffeine and alcohol for 24 h before the experiment and on the experimental days.

### Auditory Stimuli

The stimuli consisted of two categories: emotional syllables and acoustically matched nonvocal sounds. For emotional syllables, a female speaker from a performing arts school produced meaningless syllables *dada* with two sets of emotional (happy, fearful) prosodies (see Cheng et al., 2012; Hung et al., 2013; Chen et al., 2015 for validation). Emotional syllables were edited to become equally long (550 ms) and loud (min: 57 dB; max: 62 dB; mean 59 dB) using Cool Edit Pro 2.0 and Sound Forge 9.0. Each syllable set was rated for emotionality on a 5-point Likert-scale. For the fearful set, 120 listeners classified each stimulus from “extremely fearful” to “not fearful at all”. For the happy set, listeners classified from “extremely happy” to “not happy at all”. These listeners did not overlap with the participants recruited in the ERP experiment. Two emotional syllables, which were consistently identified as “extremely fearful” and “extremely happy”, were selected as the stimuli. The ratings of happy and fearful syllables on the Likert-scale (mean ± SD) were 4.34 ± 0.65 and 3.93 ± 0.97, respectively.

**Figure 1:**
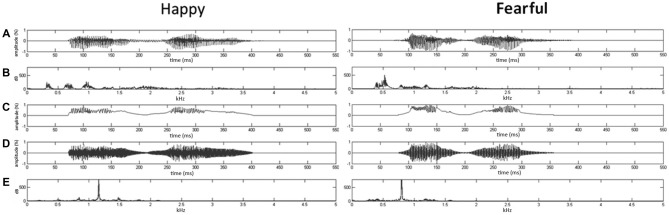
**Oscillogram and spectrogram of emotional (happy and fearful) syllables and nonvocal sounds. (A)** Oscillogram of emotional syllables [*x* axis: time (ms); *y* axis: amplitude (%)]. **(B)** Spectrogram of emotional syllables [*x* axis: kHz; *y* axis: dB]. **(C)** Sound envelop of emotional syllables and nonvocal sounds [*x* axis: time (ms); *y* axis: amplitude (%)]. **(D)** Oscillogram of nonvocal sounds [*x* axis: time (ms); *y* axis: amplitude (%)]. **(E)** Spectrogram of nonvocal sounds [*x* axis: kHz; *y* axis: dB]. Nonvocal sounds retain the spectral centroid (fn) as well as the temporal envelop of emotional syllables. The fn of fearful and happy syllables was 797.2 Hz and 1159.27 Hz, respectively.

Given that firmly controlling the spectral power distribution might cause the loss of temporal flow associated with formant contents in voices (Belin et al., 2000), the synthesis based on the temporal envelope and core spectral elementals of voices could enable to reach the maximal control of temporal and spectral features (Remedios et al., 2009). Here, using Boersma (2001) and MATLAB (The MathWorks, Inc., MA, USA), we synthesized nonvocal sounds that retained acoustic correspondence with emotional syllables. The central gravity of frequency (fn) of each original syllable was defined as [∫ |X(f)|^2^ × f *d*f/∫ |X(f)|^2^ *d*f], where X(f) was the Fourier spectrum of emotional syllables. The fn of fearful and happy syllables was 797.2 Hz and 1159.27 Hz, respectively. The nonvocal sounds were then produced by multiplying the sine waveform with two Hamming windows, which were temporarily centered at each of the syllable [nonvocal sounds = fn(t) × Hamming window(t)]. In this way, nonvocal sounds had been used for controlling the temporal envelope and core spectral element of emotional syllables (Fan et al., 2013; Chen et al., 2014; Hung and Cheng, 2014). The time-course and frequency spectrum of emotional syllables and corresponding nonvocal sounds are illustrated in Figure 1. In addition, one of previous studies using the same stimuli demonstrated that emotional syllables, rather than nonvocal sounds, exerted above-chance hit rates on the emotional categorization task, indicating emotional neutrality of acoustic controls (Chen et al., 2014).

### Procedures

Before EEG recordings, each participant completed a Chinese version of the Pittsburgh Sleep Quality Index (CPSQI; Buysse et al., 1989; Tsai et al., 2005) and the Epworth Sleepiness scale (CESS; Johns, 1991; Chen et al., 2002). The CPSQI and CESS are self-reported questionnaires that are used to assess sleep quality and to evaluate the degree of somnolence, respectively.

The subjects arrived at approximately 10:00 PM on two consecutive nights to participate in two experimental sessions, respectively: emotional syllables and nonvocal sounds. The order of emotional and nonvocal sessions was randomized and counterbalanced across subjects. We conducted EEG recordings in the examining room of Wang-Fang Hospital, where there was a separate double-walled and sound-attenuated testing chamber.

Each experimental session contained one waking block and one sleeping block for comparisons. During the waking block (11:00 PM to 12:00 AM), subjects were instructed to watch a silent movie with subtitles while task-irrelevant emotional syllables or nonvocal sounds in oddball sequences were presented. The stimulus presentation of the sleeping block was absolutely comparable with the waking block except for no need to watch a silent movie during sleep. Next, the subjects had to go to sleep for 6 h before 12:00 AM with the light off. During the sleeping block, we recorded EEG for an entire night with the auditory stimuli playing continuously. Particularly, instead of presenting physically identical stimuli as both of standards and deviants (Schirmer et al., 2007), we used the same theorem as previous works for the control of the mismatch paradigm (Čeponiene et al., 2003; Chen et al., 2014). The passive oddball paradigm for emotional syllables employed happy syllables as standards and fearful syllables as deviants. The corresponding nonvocal sounds were applied in the same oddball paradigm, but were presented as the separated session so that relative acoustic features between standards and deviants were controlled across sessions. During each block, 80% of the auditory stimuli were happy syllables or happy-derived sounds, and the remaining 20% were fearful syllables or fearful-derived sounds. The deviants ran at a random order of the sequence, edited by MATLAB (The MathWorks, Inc., USA). A minimum of two standards was always presented between any two deviants. The stimulus-onset asynchrony was 1200 ms, including a stimulus length of 550 ms and an inter-stimulus interval of 650 ms.

During the sleeping block, we recorded EEG for an entire night with the auditory stimuli playing continuously. On the following morning, immediately after waking up, participants were asked whether they had or had not consciously heard any sound during sleep, to ensure they were unaware of the sounds during sleep. The recorded lengths of the sleeping session ranged from 299.5 to 366.5 min (mean = 343.5 ± 19.5 min).

### Apparatus and Recordings

We applied compatible electroencephalography (EEG) and polysomnography (PSG) systems to record auditory ERPs and to monitor sleep, respectively. We continuously recorded EEG at 600 Hz (band-pass 0.1–100 Hz) by using four electrodes (F3, Fz, F4, and Cz) mounted on an elastic cap in accordance with a modified 10–20 system, with the addition of two mastoid electrodes (A1, A2) used as a reference, and a ground electrode placed on the forehead. Eye blinks and vertical eye movements were monitored using two electro-oculogram (EOG) electrode pairs located vertically above vs. below the left eye and horizontally at the outer canthi of both eyes. Electrode/skin impedance was maintained at <10 kΩ. EEG was epoched to 600-ms trials, including a 100 ms prestimulus baseline. Trials containing changes exceeding ±70 μV at recording electrodes and exceeding 100 μV at the EOG channels were excluded by an automatic rejection system. Trials with visually identified K-complexes that exceeded ±120 μV were also removed (Cote, 2002). We ensured the quality of ERP traces through thorough visual inspection of the data from every subject and from every trial by applying appropriate digital, zero-phase shift band-pass filtering (0.1–50 Hz, 24 dB/octave). ERP traces confirmed that muscle artifacts insignificantly contaminated all the electrodes. Submental electromyography (EMG) consisted of two electrodes placed on each side of the geniohyoid muscle with impedance maintained at <10 kΩ, which was crucial for correctly identifying REM sleep, because the waveform during REM sleep was highly similar to that during wakefulness. The electrocardiogram (ECG) consisted of two electrodes placed beneath clavicles with their impedance at 30 kΩ, for recording heart rate variability, which assisted with sleep stage scoring. We processed and analyzed ERPs using Neuroscan 4.3 (Compumedics Ltd., Australia). Notably, we applied comparable setting to record, pre-process, and segment the data during the waking and sleeping blocks for further analysis.

### Sleep Stages Scoring

The scoring of the sleep stages involved the standard scoring manual (Rechtschaffen and Kales, 1968). We recorded, amplified, digitized, and filtered the PSG with polysomnography (MedCare, USA; 27 channels) by using a ground electrode placed at Cz. According to the standard sleep-staging criteria (Rechtschaffen and Kales, 1968), successive 30-s epochs of polysomnographic data were double-blind classified by two experienced sleep technologists into five various sleep and waking stages [wakefulness, Sleep Stage 1, Sleep Stage 2, slow-wave sleep stage (combined Sleep Stages 3 and 4), and REM]. The mean heart-rate values dropped from wakefulness, light sleep, to deep sleep. During REM sleep, heart rate increased again showed a high variability, which might exceed the variability observed during wakefulness (Zemaityte et al., 1986). Using spectral analysis on heart rate variability, specific frequency ranges attributed to sympathetic and parasympathetic activities in relation with the stage changes were identified (Akselrod et al., 1981; Zemaityte et al., 1986; Berlad et al., 1993).

### Statistical Analysis

The EEG signals in the lateral electrodes (F3 and F4) relative to midline electrodes (Fz and Cz) was noisier and contaminated by the motion artifact to a larger degree due to non-conscious movements during sleep. Based on previous literatures that showed the largest effect of MMN, we analyzed the amplitudes of MMN and PMP as an average within a 100-ms time window surrounding the peak latency at the electrode sites, Fz and Cz (Näätänen et al., 2007). We defined the MMN peak as the largest negativity in the subtraction between the deviant and standard sound ERPs, during a period from 150 to 300 ms during wakefulness and from 100 to 250 ms during sleep. The N1-P2 complex was the peak-to-peak amplitude of N1 and P2 components. The PMP peak was the largest positivity within the period of 300−500 ms during wakefulness and 250−450 ms during sleep. We conducted statistical analyses, separately for experimental sessions (emotional syllables or nonvocal sounds), used a two-way repeated-measure analysis of variance (ANOVA) with stage (wakefulness, Stage 1, Stage 2, Stage 3, and REM) and electrode (Fz, Cz) as the within-subject factors. The dependent variables were the amplitudes and peak latencies of the N1-P2 complex, MMN, and PMP components at the selected electrode sites. Statistical power (1−β) was estimated by G*Power 3.1 tests (Faul et al., 2009). Degrees of freedom were corrected using the Greenhouse-Geisser method. *Post hoc* analyses were conducted only when preceded by significant main effects.

## Results

### Psychological Measures

Regarding the CPSQI, participants had optimal sleep qualities as part of their daily routine (mean score 5.3 ± 2.2). The average CESS score was 9.9 ± 3.9, indicated that the level of sleepiness during their daily routine was within the normal range.

### Sleep Staging

Table 1 displays the averaged total recording time, average time spent sleeping, average time required for initiating sleep (sleep onset latency), sleep efficiency, and duration of each sleep stage between the experimental sessions of emotional syllables and nonvocal sounds. There was no significant difference in the total recording time [*F*_(1,11)_ = 0.25, *p* = 0.63, $\eta_{p}^{2}$ = 0.022, estimated power (1−β) = 10.22%], total sleep time [*F*_(1,11)_ = 0.38, *p* = 0.55, $\eta_{p}^{2}$ = 0.033, (1−β) = 13%], sleep onset latency [*F*_(1,11)_ = 0.85, *p* = 0.38, $\eta_{p}^{2}$ = 0.072, (1−β) = 23.52%], and sleep efficiency [*F*_(1,11)_ = 0.18, *p* = 0.68, $\eta_{p}^{2}$ = 0.016, (1−β) = 8.75%] between the emotional and nonvocal sessions. We calculated the sleep efficiency of each subject based on the duration of total sleeping time divided by the total recording time. The sleep efficiency was high, with no significant differences between the emotional session (90.58%) and nonvocal session (89.58%).

**Table 1:** **Sleep staging and various measures during each experimental session**.

Sleep parameters	Emotional syllables	Nonvocal sounds	*p*-value
Total recording time (min)	344.5 ± 15.0	342.1 ± 23.3	0.63
Total sleep time (min)	312.0 ± 20.8	306.4 ± 28.3	0.55
Sleep onset latency (min)	6.5 ± 4.1	7.7 ± 5.6	0.38
Sleep efficiency (%)	90.6 ± 5.6	89.6 ± 5.7	0.68
Averaged duration (min)			
Stage 1	45.9 ± 17.3	41.8 ± 18.7	0.36
Stage 2	164.5 ± 19.9	155.8 ± 32.3	0.20
Slow wave	46.1 ± 19.1	47.8 ± 24.6	0.44
REM sleep	55.4 ± 12.9	61.0 ± 18.5	0.33

The duration of each sleep stage revealed no significant difference between the emotional and nonvocal sessions [*F*_(3,33)_ = 1.03, *p* = 0.38, $\eta_{p}^{2}$ = 0.086, (1−β) = 34.59%]. Stage 2 was significantly longer than other Stages [*F*_(3,33)_ = 87.06, *p* < 0.001, $\eta_{p}^{2}$ = 0.89, (1−β) ≈ 100%].

### Neurophysiological Measures of Emotional Salience Processing during Sleep Stages

The accepted numbers of standard and deviant trials between emotional syllables and nonvocal sounds did not significantly differ in wakefulness [happy (standard): 721 ± 209; happy-derived (standard): 707 ± 63; fearful (deviant): 180 ± 53; fearful-derived (deviant): 177 ± 18], Stage 1 (1536 ± 581; 1485 ± 585; 386 ± 150; 328 ± 150), Stage 2 (5492 ± 783, 4682 ± 1634; 1384 ± 202; 1199 ± 427), Stage 3 (1175 ± 346; 1001 ± 448; 293 ± 90; 249 ± 118), and REM (1827 ± 375; 1941 ± 631; 458 ± 95; 484 ± 157).

**Figure 2:**
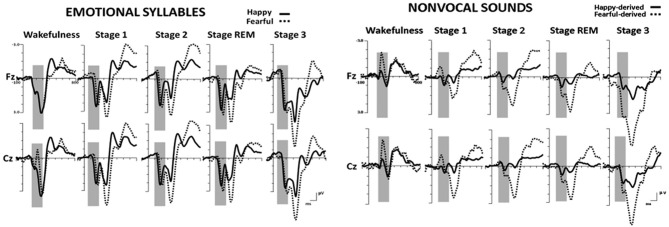
**Grand average standard (happy) and deviant (fearful) event-related potentials (ERP) waveforms in response to emotional syllables and nonvocal sounds at each sleep stage**.

**Figure 3:**
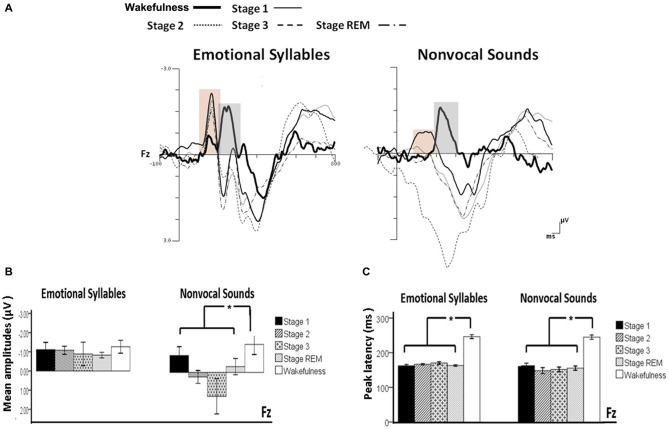
**Mismatch negativity (MMN) in response to emotional syllables and nonvocal sounds at each stage of sleep.** Emotional MMN during wakefulness was clearly detected during all sleep stages (*p* = 0.59), whereas nonvocal MMN during wakefulness was reduced at various sleep stages (*p* = 0.044). Regardless of emotional syllables (*p* < 0.001) or nonvocal sounds (*p* < 0.001), all sleep stages relative to wakefulness identified earlier latencies of MMN. **(A)** Grand average MMN waveforms. For the sake of clarity, the gray and orange area highlights the time windows of MMN during wakefulness and sleep, respectively, at the electrode site Fz. The bar graphs present the **(B)** mean amplitudes and **(C)** peak latency of MMN across electrodes. *P < 0.05.

**Figure 4:**
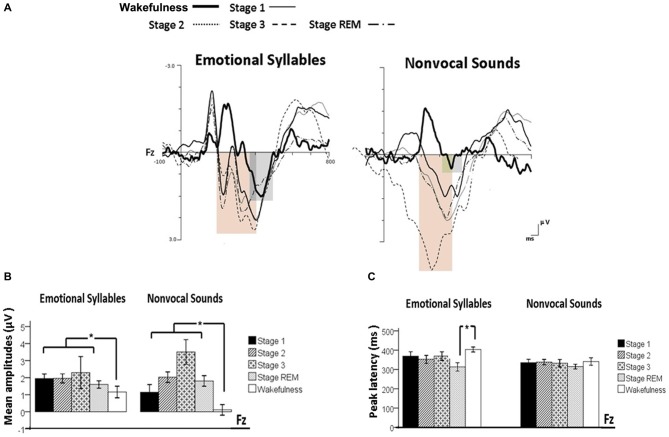
**Post-MMN positivity (PMP) in response to emotional syllables and nonvocal sounds at each stage of sleep.** Regardless of emotional syllables (*p* = 0.027) or nonvocal sounds (*p* < 0.001), all sleep stages relative to wakefulness identified stronger PMP. Emotional PMP peaked significantly earlier during rapid eye movement (REM) sleep than during wakefulness (*p* = 0.025), whereas nonvocal PMP exhibited no such a pattern (*p* = 0.07).** (A)** Grand average PMP waveform. For the sake of clarity, the gray and orange area highlights the time windows of PMP during wakefulness and sleep, respectively, at the electrode site Fz. The bar graphs present the mean amplitudes **(B)** and peak latency **(C)** of PMP across electrodes. *P < 0.05.

Figure 2 shows the ERP results for standard and deviant responses. We studied the preattentive process of emotional salience processing of voices using MMN, which was determined by subtracting happy ERP from fearful ERP (Figure 3). PMP in response to emotional syllables and nonvocal sounds at each stage of sleep are shown in Figure 4. Table 2, 3 list the mean amplitudes and peak latencies of the MMN and PMP subcomponents during each sleep stage.

**Table 2:** **Amplitudes and latencies of MMN in each sleep stage**.

		Emotional syllables		Nonvocal sounds	
MMN (Mean ± SEM)		Amplitude (μV)	Latency (ms)	Amplitude (μV)	Latency (ms)
Wakefulness	Fz	−1.26 ± 0.38	246.33 ± 5.78	−1.19 ± 0.43	244.83 ± 6.33
	Cz	−1.70 ± 0.39	230.50 ± 5.37	−1.20 ± 0.47	230.50 ± 6.04
Stage 1	Fz	−1.11 ± 0.38	162.00 ± 3.97	−0.71 ± 0.38	162.67 ± 7.06
	Cz	−0.96 ± 0.39	166.67 ± 3.51	−0.80 ± 0.37	153.00 ± 6.11
Stage 2	Fz	−1.08 ± 0.21	166.50 ± 2.35	0.19 ± 0.27	148.17 ± 8.71
	Cz	−1.04 ± 0.19	166.33 ± 2.27	0.42 ± 0.31	154.17 ± 8.39
Slow wave	Fz	−0.88 ± 0.62	170.17 ± 3.68	1.02 ± 0.75	151.67 ± 6.95
	Cz	−0.59 ± 0.44	164.00 ± 3.77	0.38 ± 0.69	163.67 ± 7.73
REM	Fz	−0.83 ± 0.14	163.00 ± 2.33	−0.25 ± 0.35	155.50 ± 6.19
	Cz	−0.68 ± 0.15	159.33 ± 3.56	−0.18 ± 0.33	165.67 ± 5.74

**Table 3:** **Amplitudes and latencies of PMP in each sleep stage**.

		Emotional syllables		Nonvocal sounds	
PMP (Mean ± SEM)		Amplitude (μV)	Latency (ms)	Amplitude (μV)	Latency (ms)
Wakefulness	Fz	1.16 ± 0.34	403.33 ± 12.94	0.11 ± 0.31	341.17 ± 19.17
	Cz	1.00 ± 0.31	402.17 ± 15.39	0.23 ± 0.33	334.00 ± 17.46
Stage 1	Fz	1.95 ± 0.27	369.17 ± 22.58	1.15 ± 0.45	335.00 ± 17.72
	Cz	2.37 ± 0.29	361.67 ± 22.44	1.80 ± 0.47	341.00 ± 17.36
Stage 2	Fz	1.96 ± 0.27	352.67 ± 20.49	2.03 ± 0.31	338.17 ± 13.91
	Cz	2.32 ± 0.36	365.33 ± 17.91	2.22 ± 0.37	347.00 ± 8.38
Slow wave	Fz	2.29 ± 0.94	369.33 ± 21.99	3.51 ± 0.72	332.33 ± 18.97
	Cz	2.24 ± 0.64	359.50 ± 22.48	2.57 ± 0.68	332.00 ± 19.11
REM	Fz	1.60 ± 0.21	313.17 ± 21.69	1.81 ± 0.31	314.83 ± 12.49
	Cz	1.73 ± 0.22	339.17 ± 17.65	1.91 ± 0.25	323.67 ± 14.33

#### The N1-P2 Complex

We subjected the N1-P2 complex amplitudes to an ANOVA regarding the stimulus (standard: happy and deviant: fearful), stage (wakefulness, Stage 1, Stage 2, Stage 3, and REM), and electrode (Fz, Cz) as repeated-measure factors for each experimental session (emotional syllables or nonvocal sounds), respectively. For emotional syllables, the ANOVA model of N1-P2 amplitudes revealed main effects of the electrode [*F*_(1,11)_ = 10.54, *p* = 0.008, $\eta_{p}^{2}$ = 0.489, (1−β) = 99.03%] and stimulus [*F*_(1,11)_ = 10.71, *p* = 0.007, $\eta_{p}^{2}$ = 0.493, (1−β) = 99.11%]. Fz had stronger N1-P2 complex than did Cz. The deviant significantly induced larger N1/P2 complex than did the standard. For nonvocal sounds, the ANOVA model of N1-P2 amplitudes showed a main effect of the stimulus [*F*_(1,11)_ = 43.14, *p* < 0.001, $\eta_{p}^{2}$ = 0.797, (1−β) ≈ 100%]. The deviant significantly induced larger N1/P2 complex than did the standard. Notably, neither emotional syllables [*F*_(4,44)_ = 1.66, *p* = 0.22] nor nonvocal sounds [*F*_(4,44)_ = 1.26, *p* = 0.31] varied N1-P2 complex among sleep stages. Both to emotional syllables and nonvocal sounds, the N1-P2 complex was undiminished from wakefulness to sleep.

#### Mismatch Negativity (MMN)

The ANOVA on MMN amplitudes to emotional syllables did not reveal any significance [stage: *F*_(4,44)_ = 0.71, *p* = 0.59, $\eta_{p}^{2}$ = 0.061, (1−β) = 26.75%; electrode: *F*_(1,11)_ = 0.15, *p* = 0.71, $\eta_{p}^{2}$ = 0.013, (1−β) = 8.6%; stage × electrode: *F*_(4,44)_ = 1.75, *p* = 0.21, $\eta_{p}^{2}$ = 0.13, (1−β) = 57.91%]. To control whether the MMN amplitude effect for fearful deviant vs. happy standard during sleep were stemming from acoustical feature differences, instead of the emotional sound content, an additional MMN analysis was conducted by subtracting the happy-derived ERP from the fearful-derived ERP. The analyses of nonvocal MMN revealed a main effect of the stage [*F*_(4,44)_ = 2.68, *p* = 0.044, $\eta_{p}^{2}$ = 0.196, (1−β) = 81.55%]. *Post hoc* analyses revealed that nonvocal MMN amplitudes were comparable at stage 1 (Bonferroni-corrected *p* = 0.52), but were reduced at stage 2 (*p* = 0.038), stage 3 (*p* = 0.082), and REM sleep (*p* = 0.041) as compared with wakefulness. These results indicated that emotional MMN could be clearly detected during all sleep stages, as well as during wakefulness. By contrast, nonvocal MMN amplitudes during wakefulness (mean ± SE: −1.19 ± 0.45 μV) were significantly decreased at sleep (Stage 2: 0.31 ± 0.28 μV; Stage 3: 0.70 ± 0.69 μV; REM: −0.22 ± 0.34 μV).

The ANOVA of MMN peak latencies to emotional syllables indicated a main effect of the stage [*F*_(4,44)_ = 118.97, *p* < 0.001, $\eta_{p}^{2}$ = 0.915, (1−β) ≈ 100%] without any effect for the electrode [*F*_(1,11)_ = 0.81, *p* = 0.39, $\eta_{p}^{2}$ = 0.069, (1−β) = 22.68%] and electrode × stage [*F*_(4,44)_ = 1.28, *p* = 0.29, $\eta_{p}^{2}$ = 0.104, (1−β) = 46.31%]. Follow-up analyses indicated that emotional MMN peak latencies during wakefulness (mean ± SE: 244.75 ± 5.23 ms) were significantly accelerated at sleep (Stage 1: 164.33 ± 2.47 ms; Stage 2: 166.42 ± 2.28 ms; Stage 3: 167.08 ± 3.02 ms; REM: 161.17 ± 2.83 ms). As for MMN peak latencies to nonvocal sounds, there were a main effect of the stage [*F*_(4,44)_ = 42.20, *p* < 0.001, $\eta_{p}^{2}$ = 0.793, (1−β) ≈ 100%] and an interaction of electrode × stage [*F*_(4,44)_ = 3.63, *p* = 0.023, $\eta_{p}^{2}$ = 0.248, (1−β) = 92.41%]. Follow-up analyses indicated that nonvocal MMN peak latencies during wakefulness (237.67 ± 5.24 ms) were accelerated at sleep (Stage 1: 157.83 ± 6.15 ms; Stage 2: 151.17 ± 7.6 ms; Stage 3: 157.67 ± 6.56 ms; REM: 160.58 ± 5.24 ms). Both Fz [*F*_(4,44)_ = 42.73, *p* < 0.001, $\eta_{p}^{2}$ = 0.795, (1−β) ≈ 100%] and Cz [*F*_(4,44)_ = 24.09, *p* < 0.001, $\eta_{p}^{2}$ = 0.687, (1−β) ≈ 100%] exhibited the stage effect, albeit with different effect size. Regardless of whether emotional syllables or nonvocal sounds were presented, MMN latencies were shortened during sleep as compared with wakefulness.

#### Post-MMN Positivity (PMP)

The ANOVA model targeting the PMP amplitudes to emotional syllables revealed a main effect of the stage [*F*_(4,44)_ = 3.03, *p* = 0.027, $\eta_{p}^{2}$ = 0.216, (1−β) = 86.53%] and an interaction of stage × electrode [*F*_(4,44)_ = 4.01, *p* = 0.007, $\eta_{p}^{2}$ = 0.267, (1−β) = 94.84%]. All of the sleep stages exhibited larger PMP amplitudes than did the wakefulness (Stage 1: 2.16 ± 0.26 μV; Stage 2: 2.14 ± 0.29 μV; Stage 3: 2.81 ± 0.57 μV; REM: 1.87 ± 0.18 μV; wakefulness: 1.08 ± 0.31 μV). *Post hoc* analysis indicated the presence of larger amplitudes for emotional PMP at sleep (Stage 2: 1.96 ± 0.27 μV; Stage 3: 3.29 ± 0.62 μV; REM: 1.93 ± 0.19 μV) than during wakefulness (1.16 ± 0.34 μV) at Fz [*F*_(4,44)_ = 4.23, *p* = 0.006, $\eta_{p}^{2}$ = 0.278, (1−β) = 95.93%], but not at Cz [*F*_(4,44)_ = 2.2, *p* = 0.08, $\eta_{p}^{2}$ = 0.16, (1−β) = 69.93%]. For the PMP amplitudes to nonvocal sounds, there was a main effect of the stage [*F*_(4,44)_ = 8.31, *p* < 0.001, $\eta_{p}^{2}$ = 0.430, (1−β) = 99.97%] and an interaction of stage × electrode [*F*_(4,44)_ = 5.92, *p* = 0.001, $\eta_{p}^{2}$ = 0.350, (1−β) = 99.39%]. PMP had larger amplitudes at sleep (Stage 1: 1.47 ± 0.45 μV; Stage 2: 2.12 ± 0.33 μV; Stage 3: 3.04 ± 0.66 μV; REM: 1.86 ± 0.28 μV) than during wakefulness (0.17 ± 0.32 μV). *Post hoc* analysis indicated that both Fz [*F*_(4,44)_ = 10.08, *p* < 0.001, $\eta_{p}^{2}$ = 0.478, (1−β) ≈ 100%] and Cz [*F*_(4,44)_ = 5.83, *p* = 0.001, $\eta_{p}^{2}$ = 0.346, (1−β) = 99.31%] exhibited a stage effect, albeit with different effect size. Regardless of whether emotional syllables or nonvocal sounds were presented, all sleep stages relative to wakefulness augmented PMP at electrode Fz.

When exploring the PMP peak latencies to emotional syllables, we observed a main effect for the stage [*F*_(4,44)_ = 3.09, *p* = 0.025, $\eta_{p}^{2}$ = 0.219, (1−β) = 87.19%]. Follow-up analyses indicated that emotional PMP peaked significantly earlier during stage 2 (359 ± 18.13 ms) and REM sleep (326.17 ± 18 ms) than during wakefulness (402.75 ± 14.08 ms). However, the PMP peak latencies to nonvocal sounds did not reveal any significance [stage: *F*_(4,44)_ = 0.531, *p* = 0.71, $\eta_{p}^{2}$ = 0.046, (1−β) = 20.42%; electrode: *F*_(1,11)_ = 1.2, *p* = 0.30, $\eta_{p}^{2}$ = 0.09, (1−β) = 28.63%; stage × electrode: *F*_(4,44)_ = 0.71, *p* = 0.59, $\eta_{p}^{2}$ = 0.061, (1−β) = 26.75%].

## Discussion

In this study, we aimed to investigate how emotional salience was processed during various sleep stages. We measured MMN and PMP, which were considered as the index of emotional salience processing and attention switching, used a passive oddball paradigm with emotional syllables along with corresponding acoustic controls, and recorded EEG during an entire undisturbed night of sleep. The results indicated that emotional MMN were clearly detected at all sleep stages, whereas nonvocal MMN was diminished during Stage 2, Stage 3, and REM sleep. The N1-P2 complex was stronger when responding to emotional syllables than to nonvocal sounds. Regardless of emotional syllable or nonvocal sounds, falling asleep from wakefulness accelerated MMN latencies and enhanced PMP amplitudes. Specifically, emotional PMP showed larger amplitudes during Stage 3 and earlier latencies during REM sleep relative to wakefulness, whereas nonvocal PMP exhibited no such pattern. The findings suggested that all sleep stages should be able to process emotional salience.

The N1-P2 complex was identified at all sleep stages, supporting the hypothesis that the sleeping brain was able to process auditory stimuli. The generator of the N1-P2 complex was presumably a network of neural populations in the primary and secondary auditory cortex (Eggermont and Ponton, 2002). The N1-P2 complex was believed to reflect the intensity of simple tones at Stage 2 (Liu and Sheth, 2009), cortical arousal (Bastien et al., 2008), and sensory sensitivity associated with involuntary orienting during REM sleep (Atienza et al., 2001b). In a case where the sleeping brain continued evaluating auditory salience (Perrin et al., 1999; Pratt et al., 1999), the N1-P2 complex during sleep was reported to be stronger in response to emotional syllables than to nonvocal sounds.

Remarkably, we detected MMN in response to emotional syllables at all sleep stages, whereas MMN in response to nonvocal sounds, as acoustic controls, was diminished during Stage 2, Stage 3, and REM sleep. Our findings in the wakefulness concurred with previous findings that were obtained with a similar paradigm in healthy awake adults (Cheng et al., 2012; Fan et al., 2013; Hung et al., 2013; Chen et al., 2014; Fan and Cheng, 2014; Hung and Cheng, 2014), indicated the validity of the current experimental design to examine the emotional processing during sleep stages. It was evidenced by that the MMN amplitude was decreased by increasing the deviant-stimulus probability, but not by the amount of deviant-stimulus *per se* (Näätänen et al., 2007). Furthermore, considering that affective discrimination was selectively driven by voice processing rather than low-level acoustical features, we hypothesized that emotional salience processing should be underpinned by cerebral specialization for human voices. The detection of nonvocal MMN at Stage 1 corroborated the automatic detection of sound changes during sleep (Ruby et al., 2008). Sleep relative to wakefulness rendered resource reallocation to alter brain activity, such as, focalized sensory cortical activation along with limited distant interaction with prefrontal cortices (Maquet, 2000; Portas et al., 2000; Drummond et al., 2004; Kaufmann et al., 2006). The MMN and PMP attenuation to nonvocal sounds during sleep might be in line with the altered neural responses to nonconsciously perceived acoustical features (Palva et al., 2005).

Emotional MMN was identified not only in REM sleep but also in the other stages of sleep, indicated that the processing of emotional salience might continue during the entire night of sleep. A general consensus seemed to support that REM sleep had a decisive role in the formation of emotional memory (Wagner et al., 2001; Hu et al., 2006; Holland and Lewis, 2007; Nishida et al., 2009). Nocturnal sleep rich in REM sleep had a priming-like enhancement of emotional reactivity (Wagner et al., 2002). REM sleep de-potentiated the amygdala reactivity to previous emotional experiences (van der Helm et al., 2011). It is worth to mention that the amygdala was activated by using a similar oddball paradigm on the perception of emotional syllables in healthy awake adults (Schirmer et al., 2008). The use of the same stimuli in an oddball paradigm indicated that the mismatch response to angry and fearful syllables was identified in sleeping human neonates (Zhang et al., 2014). Acute testosterone effect on emotional MMN further suggests the involvement of amygdala in the automatic stage of emotional salience processing (Chen et al., 2015). Along with the absence of nonvocal MMN during REM sleep, the present results demonstrated that emotional MMN during sleep stages should be selectively driven by emotional salience *per se*, rather than by acoustic changes.

Emotional PMP peaked earlier during REM sleep relative to wakefulness, whereas nonvocal PMP exhibited no such pattern. The PMP most likely reflected automatic attention orienting toward the salient deviants (Friedman et al., 2001), associated with an active processing of the deviant tone, supposedly as part of dream consciousness (Ruby et al., 2008). As expected from previous results regarding simple tones, the PMP was present during Sleep Stage 1 (Bastuji et al., 1995; Cote, 2002), 2 (Ruby et al., 2008), and REM sleep (Niiyama et al., 1994; Bastuji et al., 1995; Sallinen et al., 1996; Perrin et al., 1999; Pratt et al., 1999; Cote, 2002). Remarkably, during REM sleep, emotional PMP had the shortest latency. One explanation to conciliate previous mixed PMP findings during sleep might be that emotional syllables used in previous studies, such as the subject’s name (Perrin et al., 1999; Pratt et al., 1999), rendered the deviant stimuli particularly salient to eliciting PMP, and might not always have been based on a stimulus with less salience, such as simple tones (Bastuji et al., 1995; Sabri et al., 2000). Another possibility was explained by our increased sensitivity and specificity of PMP detection by collecting a large amount of data and by combining emotional syllables with correspondingly acoustic controls (293 ± 90 and 458 ± 95 emotional deviants, and 249 ± 118 and 484 ± 157 nonvocal deviants recorded at Sleep Stage 3 and during REM sleep, respectively). This strategy made it possible to identify a PMP component that was too weak to be detected by previous studies. Furthermore, Stage 3 was known as slow-wave sleep, whereby the sleepers tended to be unresponsive to numerous environmental stimuli. The stimuli with affective significance were likely to activate the amygdala to a greater extent during non-REM sleep (Portas et al., 2000). Supporting this, emotional (fearful vs. happy) syllables rather than nonvocal sounds elicited larger PMP values at Stage 3 relative to during wakefulness. Evolutionarily, emotional voices automatically captured attention, even during deep sleep, which might be related to survival.

Importantly, the presentation of non-awaking voices to sleeping subjects and the recording of neural dynamics elicited by emotional saliency changes help us to clarify the following. First, emotional MMN detected during sleep functionally appears equivalent to its waking counterpart. From wakefulness into sleep, accelerated MMN latencies and exaggerated PMP amplitudes may be as a result of resource reallocation. Considering the major generation of MMN with signal maxima over bilateral supratemporal cortices (Näätänen et al., 2007), reallocated focal activation within auditory cortex might enhance not only acoustic feature detection but also emotional salience processing. Second, emotional salience processing happens during every stage of sleep. The presence of double peaks within the time window of emotional MMN during sleep (please see Figure 2) can be attributed to the dissociation of two subcomponents, namely, the processes of acoustic feature and emotional salience, respectively. However, this remains future areas of inquiry. Finally, the adjacent and intensive auditory-limbic connectivity provided a platform for acoustic experience to induce structural or functional changes in the corresponding cortices (Kraus and Canlon, 2012), which, in turn, boosted automatic processing of emotional salience processing in all stages of sleep.

In conclusion, our study provides electrophysiological evidence for the processing of emotional salience during entire night of sleep. MMN in response to emotional (happy vs. fearful) syllables was detected at all sleep stages. Emotional syllables elicited stronger N1-P2 complexes than did corresponding nonvocal sounds. Emotional PMP was identified with larger amplitudes at Stage 3, and at earlier latencies during REM sleep relative to wakefulness.

## Author Contributions

CC, J-YS, and YC took part in designing the study experimental design. CC and YC undertook data analysis. CC and YC managed the literature search and wrote the first draft of the manuscript. All authors have contributed and approved the manuscript.

## Conflict of Interest Statement

The authors declare that the research was conducted in the absence of any commercial or financial relationships that could be construed as a potential conflict of interest.
